# Two-Year Outcomes of Cochlear Implant Use for Children With Unilateral Hearing Loss: Benefits and Comparison to Children With Normal Hearing

**DOI:** 10.1097/AUD.0000000000001353

**Published:** 2023-03-07

**Authors:** Lisa R. Park, Margaret T. Dillon, Emily Buss, Kevin D. Brown

**Affiliations:** Department of Otolaryngology/Head and Neck Surgery, University of North Carolina at Chapel Hill, North Carolina, USA.

**Keywords:** Binaural hearing, Cochlear implantation, Localization, Pediatrics, Single-sided deafness, Spatial hearing, Unilateral hearing loss

## Abstract

**Objectives:**

Children with severe-to-profound unilateral hearing loss, including cases of single-sided deafness (SSD), lack access to binaural cues that support spatial hearing, such as recognizing speech in complex multisource environments and sound source localization. Listening in a monaural condition negatively impacts communication, learning, and quality of life for children with SSD. Cochlear implant (CI) use may restore binaural hearing abilities and improve outcomes as compared to alternative treatments or no treatment. This study investigated performance over 24 months of CI use in young children with SSD as compared to the better hearing ear alone and to children with bilateral normal hearing (NH).

**Design:**

Eighteen children with SSD who received a CI between the ages of 3.5 and 6.5 years as part of a prospective clinical trial completed assessments of word recognition in quiet, masked sentence recognition, and sound source localization at regular intervals out to 24-month postactivation. Eighteen peers with bilateral NH, matched by age at the group level, completed the same test battery. Performance at 24-month postactivation for the SSD group was compared to the performance of the NH group.

**Results:**

Children with SSD have significantly poorer speech recognition in quiet, masked sentence recognition, and localization both with and without the use of the CI than their peers with NH. The SSD group experienced significant benefits with the CI+NH versus the NH ear alone on measures of isolated word recognition, masked sentence recognition, and localization. These benefits were realized within the first 3 months of use and were maintained through the 24-month postactivation interval.

**Conclusions:**

Young children with SSD who use a CI experience significant isolated word recognition and bilateral spatial hearing benefits, although their performance remains poorer than their peers with NH.

## INTRODUCTION

Children with unilateral hearing loss (UHL) experience difficulty with speech recognition in noise (Reeder et al. 2015; Sangen et al. 2017; Griffin et al. 2019; van Wieringen et al. 2019), sound source localization (Newton 1983; Bess & Tharpe 1984; Johnstone et al. 2010; Hassepass et al. 2013; Sangen et al. 2017; Corbin et al. 2021), and even speech recognition in quiet (Reeder et al. 2015; Griffin et al. 2019) as compared to children with bilateral normal hearing (NH). These difficulties may have long-term impacts on communication and learning. For instance, children with UHL have an increased incidence of cognitive and communication delays as compared to their peers with NH (Ead et al. 2013; Fischer & Lieu 2014; Anne et al. 2017; Sangen et al. 2017; Yu et al. 2021a, 2021b). Children with mild-to-moderate severities of UHL can be fit with a hearing aid to provide bilateral auditory input. For children with severe-to-profound UHL, also known as single-sided deafness (SSD), hearing aid use is contraindicated (Bagatto 2020; Bagatto et al. 2021). School-aged children with SSD can be fit with contralateral-routing of signal or bone-conduction hearing aids that present sound from the side of the poorer hearing ear to the NH ear; however, these devices do not provide bilateral auditory input, and are not recommended for young children (McKay et al. 2008; Bagatto et al. 2019).

Spatial hearing abilities are limited for children with SSD due to severely restricted access to binaural cues. This results in an inability to benefit from binaural summation or binaural squelch, and a limited ability to benefit from the head shadow effect. Binaural summation is the benefit associated with access to the same target speech information from both the ears. Binaural squelch occurs when timing and level differences between ears provide cues that support selective listening based on binaural difference cues. The head provides a barrier that can result in a reduction of masker level at one ear when the noise source is on the contralateral side; this results in an improved target-to-masker ratio at the ear within the head shadow, a cue not accessible at the deafened ear of patients with SSD. For individuals with NH, masked speech recognition improves when target and masker are separated as compared to when they are colocated on the horizontal plane (Bronkhorst & Plomp 1988). This benefit, known as spatial release from masking (SRM), is not observed in listeners with SSD when the masker is at the better hearing ear. Listeners with SSD can benefit from head shadow and experience SRM when the masker is to the deaf ear, but this benefit relies on monaural (as opposed to binaural) cues.

Another spatial hearing ability that is markedly degraded in listeners with SSD is sound source localization. Children with NH use timing and level differences to locate where sound is coming from on a horizontal plane. Low frequencies deliver the majority of interaural timing differences between ears, allowing the listener to determine which side of the head the sound arrived at first. Higher frequencies provide interaural level cues due to the head shadow effect. With monaural listening, children with SSD are not able to use either of these interaural difference cues to localize sound (Kumpik & King 2019).

Spatial hearing skills emerge over time in children with NH. Development impacts masked speech recognition abilities (Garadat & Litovsky 2007; Van Deun et al. 2009; Schafer et al. 2012; Corbin et al. 2016, 2021; Buss et al. 2019; Griffin et al. 2019) and sound source localization (Grieco‑Calub & Litovsky 2010; Johnstone et al. 2010; Lovett et al. 2012; Martin et al. 2015). Children may not achieve adult-like masked speech recognition scores until they approach the teenage years for challenging listening conditions (Corbin et al. 2016; Holder et al. 2016; Griffin et al. 2019; Leibold & Buss 2019). Localization matures sooner, with adult-like performance typically achieved by 5 to 6 years of age (Van Deun et al. 2010; Lovett et al. 2012). Children with congenital or early-onset SSD do not have access to the cues that are needed to develop these abilities.

Cochlear implantation is the only treatment for SSD that provides hearing to the affected ear and enables binaural auditory stimulation of the brain. Prospective clinical trials, case reports, and retrospective reviews have shown positive outcomes with cochlear implant (CI) use in this patient population. Studies have demonstrated that children with SSD who listen with a CI experience improved speech recognition in noise (Sladen et al. 2017; Thomas et al. 2017; Zeitler et al. 2019; Ehrmann-Mueller et al. 2020; Park et al. 2021a; Brown et al. 2022) and sound source localization (Arndt et al. 2015; Távora‑Vieira et al. 2019; Ehrmann-Mueller et al. 2020; Brown et al. 2022) when compared to monaural listening or preoperative performance. Also, studies of pediatric CI users with SSD generally report consistent device use (Hassepass et al. 2013; Beck et al. 2017; Polonenko et al. 2017; Ramos Macías et al. 2019; Ehrmann-Mueller et al. 2020; Ganek et al. 2020; Deep et al. 2021a; Rauch et al. 2021; Brown et al. 2022).

While the above studies suggest positive outcomes with CI use in children with SSD, recent systematic reviews have indicated that more studies are needed to confirm these benefits. For example, Huttunen et al. (2019) reported that high-quality studies investigating the consequences and benefits of interventions for early onset and congenital UHL are lacking. Also, a systematic review by Benchetrit et al. (2021) reported data for CI use in children with SSD and noted that studies are composed primarily of retrospective reviews and investigations with small, heterogeneous samples and inconsistent methods. Longitudinal controlled repeated-measures clinical trials are needed to provide professionals and families with evidence-based information on how to care for children with SSD.

The present prospective clinical trial began enrollment in 2017 and was designed to evaluate the benefits of CI use in preschool- and kindergarten-aged children with SSD. Changes in outcomes between the preoperative and early postactivation period for this cohort have been reported previously (Lopez et al. 2021; Park et al. 2021a; Brown et al. 2022). The 12-month outcome data for this trial suggested that CI use improves word recognition in the CI ear, localization, subjective hearing, binaural summation, squelch, the use of head shadow, SRM, and listening fatigue (Park et al. 2021a; Brown et al. 2022). The present report expands on that previous work by evaluating performance through 24 months of CI use in children with SSD. The primary aim of this study was to evaluate the development of auditory function following activation of the CI using repeated measures. Participants were evaluated both with and without the CI to differentiate the effects of the CI from task learning and/or developmental effects. A secondary aim of this study was to compare the performance of the SSD group, tested with and without their CI, with that of their peers with NH. Comparisons between the NH group and the SSD group tested unaided reveal challenges that children with SSD may experience in the monaural condition. Comparisons between the NH group and the SSD group when listening with their CI evaluates the magnitude of benefit in relation to the performance of children with NH. The overall goal of this project was to present age-controlled data on the detrimental effects that SSD has on auditory skills and how a CI can impact those disadvantages.

## MATERIALS AND METHODS

This investigator-initiated clinical trial received an Investigational Device Exemption from the Food and Drug Administration (FDA) and was approved by the Institutional Review Board at the University of North Carolina at Chapel Hill.

### Participants

#### Clinical Trial Participants (SSD Group)

Twenty children were enrolled in the clinical trial group (SSD group), and their demographic data are presented in Table 1. Inclusion criteria were as follows: a 3-frequency pure-tone average (average at 500, 1000, and 2000 Hz) of ≥70 dB HL in the affected ear, a 3-frequency pure-tone average of ≤25 dB HL in the contralateral ear, 3.5 to 6.5 years of age at the time of implantation, anatomically normal cochlear nerve, cochlear anatomy amenable to implantation (defined as no more severe than incomplete partition type II), aided consonant nucleus consonant (CNC) word recognition of ≤30% in the affected ear, typical cognition as measured by the Leiter-R test of nonverbal intelligence (Roid & Miller 2002), and parental commitment to procedures. Children were excluded if English was not their primary language, there was an untreatable conductive hearing loss in either ear, ossification was noted, or the hearing loss was sudden and had not been evaluated by a physician. Two children (participants 06 and 10) were included in the study group under FDA reviewed single-subject expanded access; these participants exceeded the age criterion but reported short durations of SSD (i.e., <3 years). Their data were omitted from the present report.

**TABLE 1.** Demographic data for the clinical trial participants

ID	Age at Surgery (yr)	Length of Deafness (yr)	Age at Diagnosis	NBHS Result	Sex	Etiology (Deafened Ear)	Stability (Deafened Ear)	Affected Ear	Pre-Op PTA CI Ear (dB HL)	Pre-Op PTA Contra Ear (dB HL)
01	6.4	1.9	4 y	Pass	Male	Infection	Known sudden	Right	112	2
02	6.3	1.4	5 y	Pass	Female	Unknown	Reported sudden	Right	88	15
03	4.5	1.4	3 y	Pass	Female	Trauma	Known sudden	Right	120	3
04	6.1	6.1	2 y	Fail, pass rescreen	Female	Malformation	Suspected congenital	Left	85	20
05	4.7	4.7	2 m	Failed unilateral	Male	Waardenburg	Known congenital	Right	118	20
07	4.0	4.0	8 m	Failed unilateral	Male	Malformation	Known congenital	Right	82	10
08	6.5	4.6	3 y	Pass	Male	Unknown	Suspected progressive	Right	120	7
09	6.5	6.5	6 y	Fail, pass rescreen	Female	Unknown	Suspected congenital	Left	110	8
11	6.1	3.5	5 y	Pass	Female	Unknown	Suspected progressive	Right	120	2
12	3.9	1.8	2 m	Failed unilateral	Female	cCMV	Known progressive	Left	113	22
13	4.8	4.2	2 y	Failed unilateral	Male	Unknown	Known progressive	Right	120	8
14	5.4	1.3	4 y	Pass	Male	Unknown	Reported sudden	Left	118	2
15	5.5	0.8	3 y	Was not tested	Male	Unknown	Known progressive	Left	77	8
16	3.7	3.8	2 y	Fail, pass rescreen	female	Unknown	Suspected congenital	Right	120	8
17	5.4	5.4	1 m	Failed unilateral	Male	Unknown	Known congenital	Left	117	8
18	3.5	3.6	2 m	Failed unilateral	Male	Unknown	Known congenital	Left	120	15
19	3.9	3.9	3 y	Fail, pass rescreen	Male	Unknown	Suspected congenital	Left	110	15
20	3.6	3.6	1 m	Failed unilateral	Male	cCMV	Known congenital	Right	120	3

Length of deafness is calculated based on the date of activation (typically 2–4 wk after surgery).

cCMV, congenital cytomegalovirus; CI, cochlear implant; dB HL, decibels hearing level; m, months; NBHS, newborn hearing screen; PTA, pure-tone average (average at 500, 1000, and 2000 Hz); yr, years.

published online ahead of print May 7, 2023.

Most of the participants with SSD had a severe-to-profound hearing loss in the affected ear (n = 16); two children (participants 07 and 15) had profound rising to moderate hearing loss configurations. Length of deafness was not documented in the medical record for eight participants. Their lengths of deafness were estimated based on parental report and were included in the analysis. Ten participants were affected in the right ear and eight in the left. All had receptive and expressive language skills within the normal range at the time of surgery based on testing with the Oral and Written Language Scales, Second Edition (Carrow-Woolfolk 2011).

#### Normal Hearing Participants (NH Group)

A group of 18 children with bilateral NH (NH group: 10 male and 8 female children) were recruited for comparison and completed the study test battery at a single visit. Conventional audiometry was used to verify NH, with pure-tone stimuli presented via insert earphones. All participants had thresholds of 20 dB HL or better in both ears at each octave frequency from 125 to 8000 Hz. Children in the NH group were of similar age to the children in the SSD group at the 24-month postactivation interval (SSD *M* = 7.0 years, SD = 1.1; NH *M* = 6.6 years, SD = 1.3). Age comparisons between groups using a Mann-Whitney U test indicated that the two distributions were not significantly different (U = 201.50, *z* = 1.251, *p* = 0.214).

#### Surgery and Devices

Participants in the SSD group received a MED-EL SYNCHRONY internal device with either a FLEX28 (n = 17) or FLEX24 (n = 1) electrode array, at the surgeon’s discretion. The electrode array was fully inserted based on the surgeon report in all but one case (participant 04); that participant had a Mondini malformation and 2 extracochlear electrode contacts. The mean age at implantation was 5.0 years (SD = 1.1), with an estimated 3.5 years of deafness (SD = 1.7).

Participants in the SSD group listened with the SONNET ear-level processor. Devices were activated ~2 weeks after surgery, and mapping was completed after testing at each follow-up visit. The processor microphones were programmed for omnidirectional mode. The participants’ devices were initially programmed using behavioral methods, with most comfortable levels (MCLs) set to loud but comfortable. If behavioral measures were judged to be unreliable, the MCL levels were determined using electric stapedial reflex thresholds. MCLs were balanced in loudness to the contralateral ear when the participant was developmentally able to make these judgments. During the balancing task, MCLs were globally increased or decreased in live voice until the participant judged the loudness in the CI ear to be equal to the loudness of the ear with NH. Threshold levels were measured behaviorally with conventional or conditioned play methods for 50% detection and were then reduced by 15%. Stimuli were presented sequentially across the active channels at threshold level to confirm that they were not audible. The filter frequency range was set to 100 to 8500 Hz at activation; this range was adjusted during the study in some cases, based on the clinical judgment. Participants were tested at default volume (100%) and sensitivity (75%) settings.

#### Listening Therapy

Participants in the SSD group received auditory therapy from a listening and spoken language certified speech-language pathologist during the first year of CI use. Therapy was completed remotely using direct connection to the processor to isolate the input to the implanted ear. Sessions occurred every other week for the first 6 months and then monthly for the remainder of the first year of CI use. Formal auditory therapy was then discontinued. All participants attended all study therapy visits; however, any therapy occurring outside of the study was not tracked.

### Procedures

Testing took place in either a single-walled or double-walled sound-proof booth. A test assistant was present to keep participants focused and engaged, and breaks were provided as needed. Each test session was completed with the use of the familiar CI map. Routine mapping occurred after completion of the test battery. The NH test battery took ~45 minutes to complete, while the full SSD test battery lasted ~1 hour and 15 minutes.

#### Detection Thresholds

Audiometric thresholds were measured with insert earphones and a GSI AudioStar Pro audiometer. Procedures included play and conventional audiometry, depending on participant age and ability. The unaided thresholds for the SSD group were measured every 6 months.

#### Word Recognition

Single-word recognition testing was completed to answer two questions: (1) how does the performance of the NH group compare to the performance of the SSD group when participants listened with their NH ear alone? and (2) how does the performance change over time for the SSD group when listening with their CI alone? Performance was measured with recorded 50-item CNC word lists (Peterson & Lehiste 1962). Responses were scored as the percent of words correctly repeated.

Word recognition for the SSD group at 24-month postactivation was compared to NH performance by measuring word recognition in the sound field. Stimuli were presented at 60 dBA. The participant was seated 1 m from the speaker at 0° azimuth. Participants in the NH group were tested with both ears unoccluded (NH binaural). Participants in the SSD group were tested with the CI off and both ears unoccluded (SSD NH alone).

To assess the development of word recognition with a CI, CNC words were presented to the affected ear alone via direct audio input (DAI) to the processor at 3-, 6-, 9-, 12-, 18-, and 24-month postactivation (SSD CI-alone). DAI was chosen to isolate the CI ear from the contralateral ear, as previously described (Park et al. 2021b; Brown et al. 2022). Briefly, the SONNET FM battery sleeve was coupled to a 90/10 audio cable, which was inserted into the audio jack of the computer used for speech recognition testing. The stimuli were presented using iTunes, and the participants were allowed to adjust the volume at each visit to a comfortable level, typically 95 to 100%.

#### Masked Sentence Recognition

Masked sentence recognition testing was completed to compare the performance of the NH group to the SSD group, and to monitor the development of skills of the SSD group with a CI over time. Performance was evaluated with the Bamford-Kowal-Bench Speech-in-Noise test (BKB-SIN) (Etymotic 2005). Target stimuli were presented at 60 dBA in the sound field. The intensity of the 4-talker masker increased by 3 dB steps for each sentence in a 10-sentence half-list. Testing was carried out using two loudspeakers that were mounted in adjacent corners of the sound booth; the participant was seated 1 m from each loudspeaker, with one loudspeaker in front (0°) and the other at 90° to the left or right (King et al. 2020). Participants were tested in three target-to-masker configurations: (1) masker colocated with target (speech at 0° azimuth, masking noise at 0° azimuth (collocated) [S_0_N_0_]); (2) masker 90° to the affected ear (SSD) or left ear (NH) (speech front and masker to the CI or left ear [S_0_N_ci/L_]); and (3) masker 90° to the NH or right ear (speech front and masker to the normal hearing or right ear [S_0_N_nh/R_]). Participants in the NH group were tested in the NH binaural condition. Participants in the SSD group were tested with the NH alone and in the combined listening condition (CI+NH) at the 6-, 12-, and 24-month postactivation intervals for each target-to-masker configuration. The test assistant monitored head position and reminded participants to keep their heads facing midline for the test session. Two half-lists were given in each condition. An signal-to-noise ratio required for 50% correct was calculated per the test manual.

#### Sound Source Localization

Sound source localization was evaluated to compare the performance of the NH group to the SSD group, and to monitor the development of skills with a CI over time. Testing was completed with a 180° arc of 11 loudspeakers that were evenly spaced 18° apart (Dillon et al. 2017). The participants were seated in the center of the arc, ~1 m from the loudspeakers. Chair height was adjusted as necessary so that the participants’ ears were level with the center of the loudspeakers. A picture of an animal was placed above each loudspeaker, the room was darkened, and the participants wore a headlamp. A 200-msec speech-shaped noise burst was presented at 70 dB SPL from one of the loudspeakers, selected at random. The participant was instructed to turn their head toward the sound, lighting the animal with the light from the headlamp. The stimulus was presented from each sound source four times for a total of 44 trials. The test assistant sat under the center speaker to keep the participant engaged and direct them to return to midline after each trial. Root-mean-square error (RMS_err_) was calculated for each block of trials as described by Buss et al. (2018), along with variable and constant error as described by Grantham et al. (2007). The NH group was tested in the NH binaural condition. The SSD group was evaluated in the CI+NH and NH alone conditions at the 3-, 9-, 18-, and 24-month postactivation intervals.

#### Data Analysis

Statistical analysis was completed with R (R Core Team 2021). The nlme (Pinheiro & Bates 2000) and emmeans (Lenth et al. 2019) packages were used for analysis. F-statistics were computed for models including categorical factors with more than two levels. A pseudo R^2^ value was calculated for each model to give an estimate of effect size using the R2glmm package (Knudson et al. 2021). Percent correct CNC word scores were converted to rationalized arcsine units (Studebaker 1985) prior to analysis. Bonferroni corrections were applied for multiple comparisons when following up on interactions observed in mixed models. Results for each task were modeled twice. One model compared 24-month data from the SSD group to the NH group; this model included participant age, mean centered on 7.0 years. The other model evaluated postactivation performance in the SSD group over time; this model included the age at CI activation, mean centered on 5.0 years. Centering was achieved by subtracting the mean from each participant’s age. All models included random intercepts for each participant. There were no missing data for either group.

## RESULTS

All participants in the SSD group maintained NH thresholds in the contralateral ear throughout the course of the study apart from one participant (participant 12), who acquired a flat mild loss (PTA = 33 dB HL) by the 12-month interval. Participant 12, whose etiology was congenital cytomegalovirus, was fit with a hearing aid that was used during testing for the remainder of the study. Sex was included in preliminary statistical models and was found to be noncontributory; given that no sex effect was predicted, it was not included in the analyses reported below.

Participants in the SSD group listened with their CIs consistently, with average device use ranging from 7.1 to 12.0 hours per day. Data logs indicated that the processors were powered on a median 10.0 hours per day over the course of the study (interquartile range, 8.1–11.0 hours) and 9.2 hours per day at the 24-month postactivation interval (interquartile range, 6.8–10.2 hours).

### Word Recognition

**Fig. 1.**
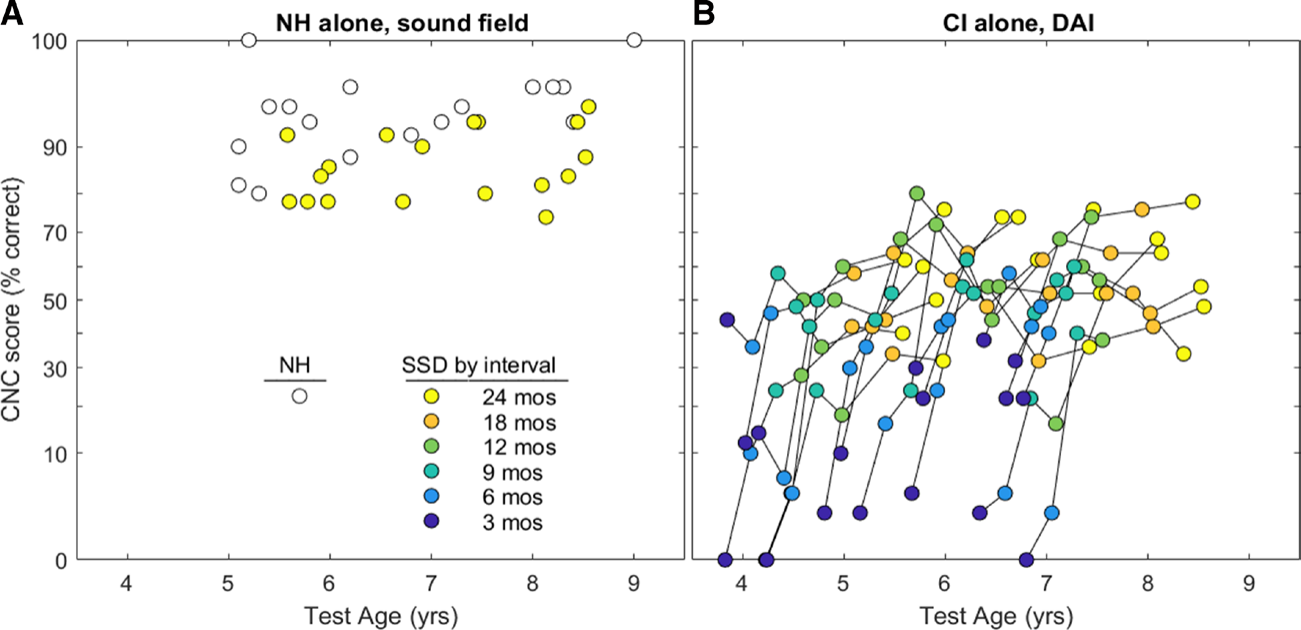
Between group comparison of the distribution of consonant nucleus consonant (CNC) word scores for the SSD and NH groups. The analysis was completed in rationalized arcsine units (RAU), and results are plotted as % correct on a RAU scale as a function of age. A, The scores for the NH group tested binaurally are presented in white, while the SSD group listening with the NH ear alone are presented in yellow. Postactivation interval is coded by color for the SSD group in (B). DAI indicates direct audio input; NH, normal hearing; SSD, single-sided-deafness.

CNC word scores obtained in the sound field are shown as a function of age in Figure 1A. The full statistical model is presented in Supplemental Digital Content 1, http://links.lww.com/EANDH/B108. The first analysis compared the performance for the NH group to the performance for the SSD group when listening with their NH ear alone at the 24-month interval. These groups were evaluated with a linear mixed model that also included age at test and a random intercept for each participant. The SSD group performance in the sound field (M = 85.7%, SD = 7.04) was significantly poorer than the NH group (M = 93.4%, SD = 5.81, *p* < 0.001). There was also a significant main effect of age (*p* = 0.026), with older children experiencing better performance than younger children. The interaction of group and age was not significant (*p* = 0.538), indicating that age affected both groups similarly.

The second analysis assessed the change in performance over time as a function of the interval while accounting for the age at activation as a covariate for the SSD group when listening with their CI alone. Figure 1B illustrates the CNC word scores measured using DAI over the study period. The full statistical model is included in Supplemental Digital Content 2, http://links.lww.com/EANDH/B108. Interval was log transformed prior to analysis. There was a significant effect of test interval (*p* < 0.001). Age at activation and the interaction between interval and age at activation did not approach significance (*p* ≥ 0.90). This indicates that improvement in CNC scores over time did not depend on the age at activation. Visual inspection of Figure 1B suggests that the largest improvements occurred within the initial months of CI use. Mean performance improved by 31% points in the first 6 months (from 14.2% correct at 3 months to 45.0% correct at 9 months), with an additional improvement of 6% points in the final 6 months (from 51.7% correct at 18 months to 57.8% correct at 24 months).

### Masked Sentence Recognition

**Fig. 2.**
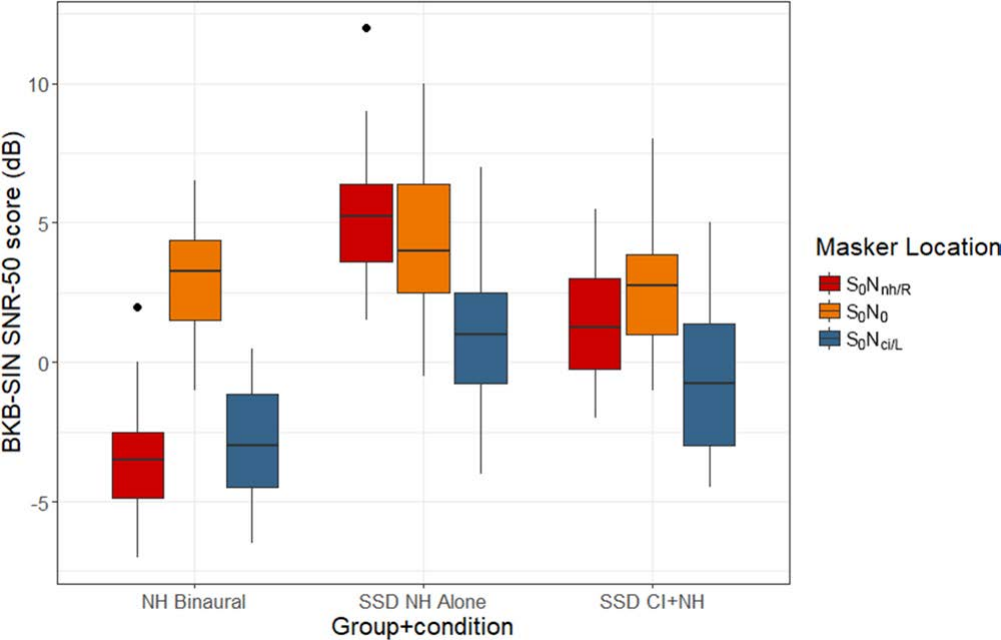
Between group comparison of Bamford-Kowal-Bench Speech-in-Noise test (BKB-SIN) scores for each group+condition, plotted as SNR-50 in dB. Masker location is indicated by color as defined in the legend. Group+condition is indicated along the horizontal axis. The center line indicates the median, the boxes span the 25th and 75th percentiles, the whiskers indicate the minimum and maximum scores within 1.5 IQR of the lower and upper quartile, and the dots are outlying points. CI indicates cochlear implant; IQR, interquartile range; NH, normal hearing; S_0_N_0_, speech front and masker front; S_0_N_nh/R_, speech front and masker to the normal hearing or right ear; S_0_N_ci/L_, speech front and masker to the CI or left ear; SNR-50, signal-to-noise ratio in dB for 50% correct; SSD, single-sided-deafness.

Figure 2 plots the masked sentence recognition performance for the NH group and the SSD group tested with the NH ear alone and in the CI+NH condition for each masker location at the 24-month interval, with lower values indicating better performance. Performance of the SSD group was compared to the NH group with a linear mixed model that included age at the test point, group and condition (SSD group with CI+NH, SSD group with NH alone, and NH group), and target-to-masker configuration (S_0_N_0_, S_0_N_nh/R_, and S_0_N_ci/L_). For this model, the SSD CI+NH group and condition and the S_0_N_0_ target-to-masker configuration were entered as the reference conditions. Comparisons of interest include the following: (1) the NH group and the SSD group with the NH ear alone, (2) the NH group and the SSD group with the CI+NH, and (3) the SSD group when listening in the CI+NH versus NH alone conditions to evaluate the benefit of CI use.

Results are reported in Supplemental Digital Contents 3 and 4, http://links.lww.com/EANDH/B108. Interactions with age at test were nonsignificant (*p* ≥ 0.579), so they were removed from the model to increase the power to observe lower-order effects. There were significant main effects of age at test [*F*(1,34) = 11.74, *p* = 0.002], indicating better performance (i.e., lower scores) for older than younger children. There were also effects of group and condition [*F*(2,118) = 5.11, *p* = 0.007], and target-to-masker configuration [*F*(2,118) = 17.14, *p* < 0.001], as well as significant interactions between target-to-masker configuration and the combination of group and condition [*F*(4,118) = 23.01, *p* < 0.001]. Estimated marginal means indicate that there was no significant difference between the NH group and the SSD group in either the CI+NH and NH alone conditions when speech and masker were colocated (*p* ≥ 0.083). The NH group performed significantly better than the SSD group when the masker was directed to the CI/left ear in both the CI+NH (*p* = 0.002) and NH alone conditions (*p* < 0.001). The same pattern was noted when the masker was directed to the NH/right ear. The NH group had significantly better scores than the SSD group both in the CI+NH (*p* < 0.001) and NH alone conditions (*p* < 0.001). When probing the difference in BKB-SIN scores in the CI+NH versus NH alone conditions for the SSD group, the use of a CI improved performance in all three target-to-masker configurations (S_0_N_0_, *p* = 0.013; S_0_N_ci/L_, *p* = 0.027; S_0_N_nh/R_, *p* < 0.001).

Together these results indicate that the SSD group performed similarly to the NH group in the colocated condition, both with and without the CI. While the CI significantly improved performance for the SSD group in all target-to-masker configurations, performance was still poorer than the NH group in both spatially separated target-to-masker configurations when tested in the CI+NH condition. For reference, the NH group had an average SRM of 6.0 dB with the masker on either side. For the SSD group, the average performance in the CI+NH condition was 3.2 and 1.3 dB with the masker to the CI and NH ears, respectively. With the NH ear alone, SRM was 3.4 dB in the S_0_N_ci_ condition and −1.1 dB with the masker to the NH ear, with findings in the speech at 0° azimuth, masking noise at +90° azimuth facing the ear with normal hearing (S_0_N_nh_) condition indicating a detrimental effect of spatial separation.

**Fig. 3.**
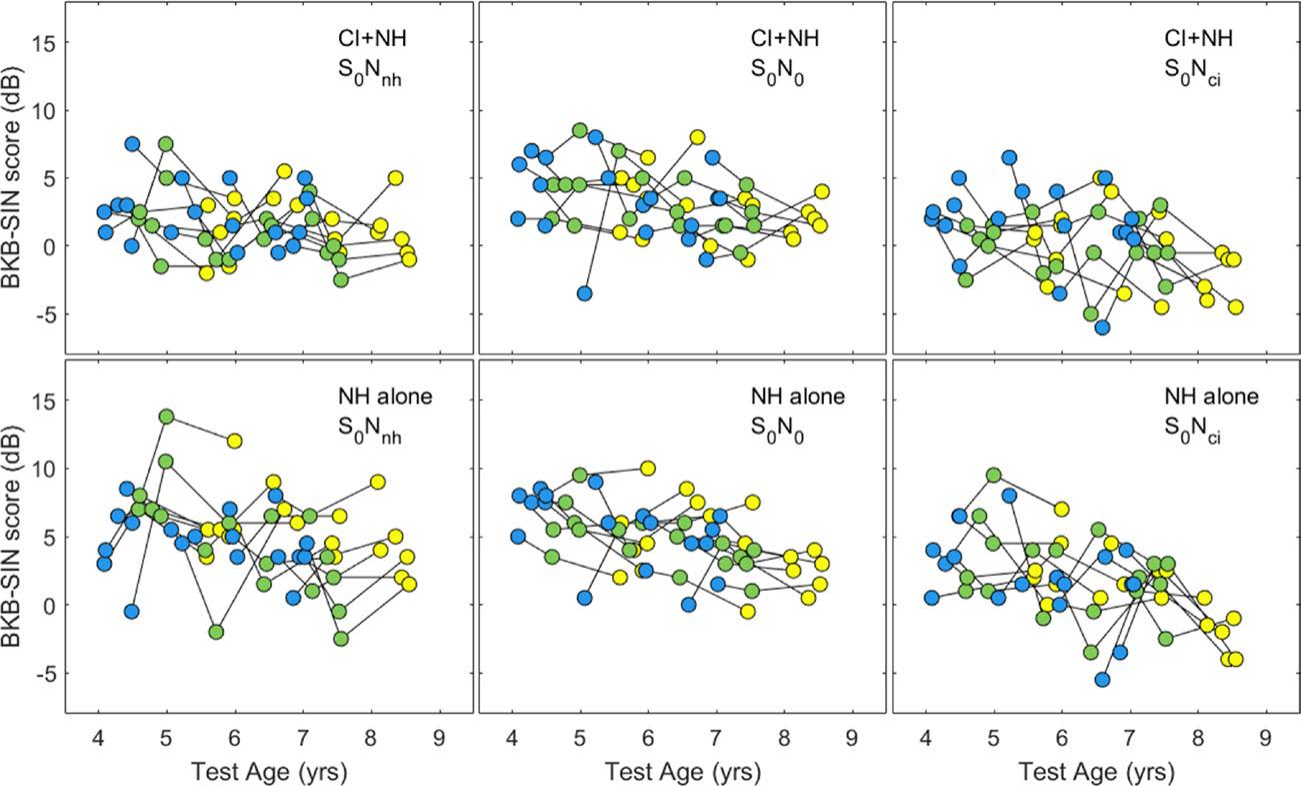
Within group comparison of the distribution of Bamford-Kowal-Bench Speech-in-Noise test (BKB-SIN) scores for the SSD group (SNR-50), plotted as a function of age for each condition of device use and masker location. Postactivation interval is indicated by the same colors used in Figure 1. CI indicates cochlear implant; S_0_N_0_, speech front and masker front; S_0_N_ci_, speech front and masker to the CI ear; S_0_N_nh_, speech front and masker to the NH ear; SNR-50, signal-to-noise ratio in dB for 50% correct; SSD, single-sided-deafness.

An additional linear mixed model evaluated the effects of interval (6-, 12-, and 24-month postactivation), condition (CI+NH versus NH alone), and target-to-masker configuration (S_0_N_0_, S_0_N_nh_, and S_0_N_ci_) on the development of masker sentence recognition skills in the SSD group, with age at CI activation included in the model. These scores are plotted in Figure 3. Results of the analysis are in Supplemental Digital Content 5, http://links.lww.com/EANDH/B108, with comparison of EMMs reported in Supplemental Digital Content 6, http://links.lww.com/EANDH/B108. These analyses indicate a significant main effect of age at activation [*F*(1,16) = 14.89, *p* = 0.001], device [*F*(1,300) = 14.21, *p* < 0.001], target-to-masker configuration [*F*(2,300) = 29.35, *p* < 0.001], and interval [*F*(1,300) = 5.67, *p* = 0.018], as well as an interaction between device and target-to-masker configuration [*F*(2,300) = 4.25, *p* = 0.015]. In the CI + NH device condition, BKB-SIN scores improved from the S_0_N_0_ condition to each spatially separated condition, reflective of SRM. In the NH alone condition, SRM was notable when the masker was directed to the CI ear but not when the masker was directed to the NH ear. Scores improved with later ages of activation. It is possible that the age of activation acted as a surrogate for age at test in this model, as speech recognition in the presence of a masker is known to improve with age (Garadat & Litovsky 2007; Van Deun et al. 2009; Schafer et al. 2012; Corbin et al. 2016, 2021; Buss et al. 2019; Griffin et al. 2019). Including a three-way interaction with device, target-to-masker configuration, and either age at activation or test interval did not reveal any other significant effects.

### Localization

**Fig. 4.**
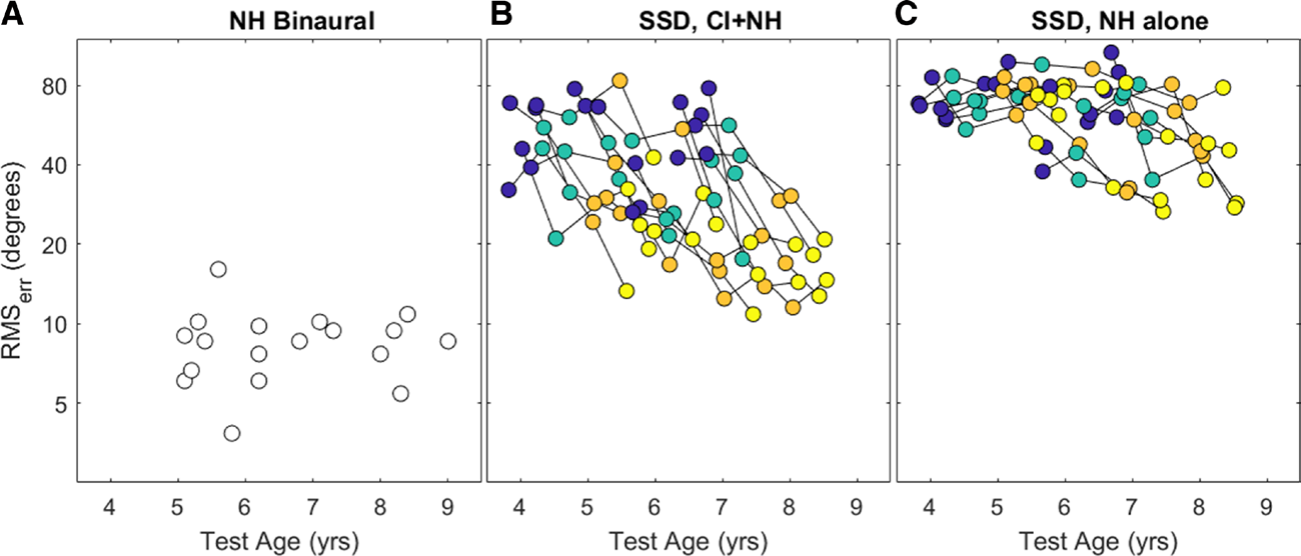
Between and within group comparisons of the distribution of localization scores. Results are plotted as root-mean-square error (RMS_err_) on a Log2 scale as a function of age. Results for the NH group are plotted in white in (A). Results for the SSD group are color coded by postactivation interval (see Fig. 1). The CI+NH condition results are shown in (B) and the NH alone results are presented in (C). CI indicates cochlear implant; NH, normal hearing; RMS, root-mean-square; SSD, single-sided-deafness.

Figure 4 plots the sound source localization performance in RMS_err_ for the NH group (Fig. 4A) and the SSD group in the CI+NH and NH alone conditions (Figs. 4B, C, respectively). The first model evaluated the main effects of age for the NH group and the SSD group at the 24-month interval; this model included age at test, the combination of group and condition (SSD group with CI+NH, SSD group with NH alone, and NH group), and the two-way interaction between these factors. RMS_err_ was logarithmically transformed prior to analysis. Results are reported in Supplemental Digital Content 7, http://links.lww.com/EANDH/B108. There was a significant main effect of group and condition [*F*(2,14) = 132.05, *p* < 0.001], but no significant main effect of age [*F*(1,34) = 0.09, *p* = 0.766]. There was a nonsignificant trend for an interaction between age and the combination of group and condition [*F*(2,14) = 3.12, *p* = 0.076]. Children in the NH group had the smallest RMS_err_ (geometric mean [GM] = 8.2°, geometric standard deviation [GSD] = 1.37), followed by the SSD group listening CI+NH (GM = 19.7°, GSD = 1.42), and finally the SSD group with the NH ear alone (GM = 49.9°, GSD = 1.52). Estimated marginal means indicate that each of these group differences was significant (*p* < 0.001). Compared to the NH group, the SSD group exhibited a more significant decrease in RMS_err_ with increasing age for the NH alone condition (*p* = 0.030), but not in the CI+NH condition (*p* = 0.087). Notably, 6 participants (01, 08, 11, 14, 15, and 18) in the SSD group were approaching NH group performance after 24 months of use in the CI+NH condition.

The RMS_err_ results over time for the SSD group in the CI+NH and NH alone conditions are plotted in Figures 4B, C, and model results are presented in Supplemental Digital Content 8, http://links.lww.com/EANDH/B108. The linear mixed model included the main effects of age at activation, interval, and device (CI on and off), as well as the associated two-way and three-way interactions. The model indicated significant main effects of device [*F*(1,120) = 231.31, *p* < 0.001], suggesting improved performance in the CI+NH condition, and a main effect of age at activation [*F*(1,16) = 5.27, *p* = 0.036]. There was a significant interaction between device and interval [*F*(1,120) = 33.20, *p* < 0.001], reflecting the fact that RMS_err_ decreased across intervals at a greater rate for the CI+NH condition than the NH alone condition. There was also an interaction between age at activation and interval [*F*(1,120) = 14.05, *p* < 0.001], reflecting the fact that increased age of activation was associated with better performance. When considering the main effects as well, localization abilities may have started at a similar level, but older children may have progressed to their plateau more quickly. As increasing age at test was associated with improved localization for the SSD group in the earlier model that compared the NH and SSD groups, it is likely that age of activation was a proxy for age at test in this model.

**Fig. 5.**
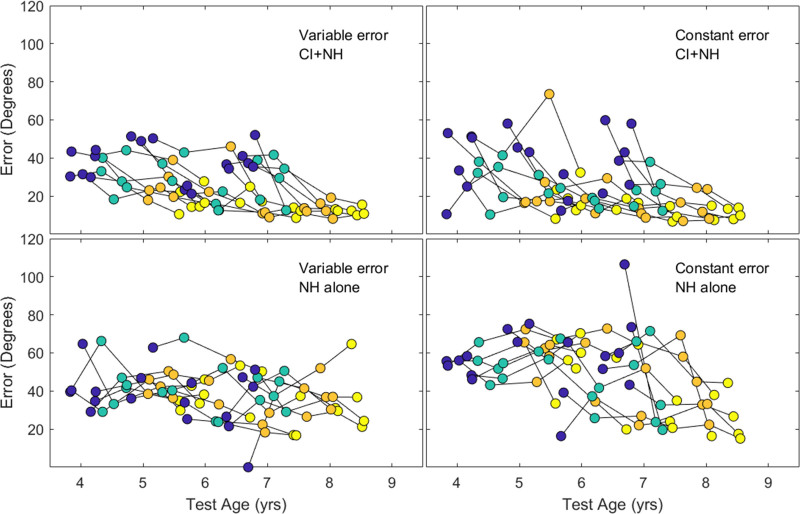
Within group comparison of the distribution of variable error (A) and (B) and constant error (C) and (D) plotted a function of age and grouped by CI+NH vs. NH alone. Postactivation interval is coded by color (see Fig. 1). CI indicates cochlear implant; NH, normal hearing.

Sound source localization was also quantified using measures of variable error and constant error with the CI+NH versus NH alone for the SSD group; results are plotted in the left panels of Figure 5 and presented in Supplemental Digital Content 9, http://links.lww.com/EANDH/B108. Variable error is the average of the SDs of the responses for each source, with lower values indicating more consistent responses. A linear mixed model evaluated effects of age at activation, test interval, and device (CI on and off) in the SSD group. In contrast to the previous analysis of RMS_err_, variable error was approximately normal without transformation. Age at activation, device, and interval were not significant main effects (*p* ≥ 0.169). Only the interaction between device and interval reached significance (*p* < 0.001), reflecting the fact that variable error improved (fell) across intervals with the CI+NH, but not with the NH ear alone.

Constant error is a measure of systematic bias in responses, with lower values indicating less of a bias to either side. It is the magnitude of the difference between the actual source location and the average of the distribution of responses. Constant error was evaluated using a similar model as described above for variable error. Results are plotted in the right panels of Figure 5 and presented in Supplemental Digital Content 10, http://links.lww.com/EANDH/B108. The main effect of age at activation was not significant (*p* = 0.281). There were significant main effects of device (*p* < 0.001) and interval (*p* = 0.001). There was a three-way interaction between age at activation, device, and interval (*p* = 0.029). Visualization of the data suggests that with the CI on, age at implantation was not a factor in the early months, but much like what was noted in the RMS_err_ analysis, participants implanted at older ages appear to have followed a steeper trajectory in later follow-up intervals. Older age at implantation (and hence older age at the time of testing) was associated with greater constant error with the NH ear alone at the 3-month interval than at later intervals.

## DISCUSSION

When compared to their peers with NH, children with SSD are at a disadvantage with regard to word recognition in quiet, masked sentence recognition, and sound source localization. The results of this study indicate that a CI can benefit children with SSD in unilateral and bilateral listening tasks, although their outcomes still lag behind those of their peers with NH. Participants in the SSD group experienced significant improvements for masked sentence recognition and sound source localization in the CI+NH condition, benefits that were observed through 24 months of CI use.

### Monaural Performance of the SSD group Versus Binaural Performance of the NH group

The results of this study show that in a controlled, quiet environment, children with SSD have poorer word recognition than their peers with NH. Reeder et al. (2015) had similar findings when investigating CNC word recognition at 50 dB SPL, compared to 60 dBA in the present study. Griffin et al. (2019) found that children with UHL had poorer performance on sentences in quiet than children with NH. These results indicate that in the most advantageous listening conditions, children with UHL do not perceive speech as well as their NH hearing peers. This could be due to a lack of summation or a subclinical hearing loss that reduces access to speech cues. In the present study, five of the participants with SSD would not have passed the same screening as the NH group in their better hearing ear, suggesting the NH group had more access to speech than the SSD group.

The results of this study corroborate others reporting that children with UHL have more difficulty perceiving speech with spatially separated maskers than children with NH. The magnitude of SRM noted in this study for the NH group (~6 dB) is similar to what has been reported in previous studies (Lovett et al. 2012; Misurelli & Litovsky 2015; Griffin et al. 2019). Previous work has also found that children with NH experience SRM when the masker is presented from either the left or right side, while those with UHL do not have SRM when the masker is at the better ear (Reeder et al. 2015; Griffin et al. 2019; Corbin et al. 2021). In the present study, the SSD group had a mean SRM of 3.4 dB when the masker was presented towards the poorer ear and a SRM of −1.1 dB when the masker was presented at the better ear. Negative values of SRM indicate that performance was worse in the S_0_N_nh_ condition than in the colocated condition for participants with SSD.

Other findings from the present study related to masked speech recognition were mixed when compared to data from the literature. As in the present report, Reeder et al. (2015) did not find a difference in scores between children with NH and those with UHL when speech was colocated with a masker composed of speech; however, Griffin et al. (2019) did find a difference. Regardless of whether the masker was separated to the side of the affected/left ear or the better hearing/right ear, NH listeners outperformed listeners with SSD in both the present study and published data (Reeder et al. 2015; Griffin et al. 2019; Corbin et al. 2021). Age effects were noted in masked sentence recognition, with older children demonstrating improved performance. Griffin et al. (2019) reported similar age effects, although Reeder et al. (2015) did not find evidence of improvement with age. It is unclear what accounts for this discrepancy in results, but it could be due to differences in the age range of participants, mean age, length of deafness, or age of onset of hearing loss.

Unsurprisingly, the present findings replicate other studies that noted poorer localization in children with SSD than in children with NH (Newton 1983; Bess & Tharpe 1984; Reeder et al. 2015; Corbin et al. 2021). For children with NH, the present study found a mean RMS_err_ of 8.2°, similar to values reported by others. For example, Reeder et al. (2015) and Corbin et al. (2021) reported mean RMS_err_ of 6° and 8.6°, respectively. For the SSD group in the present study, participants had a mean unaided RMS_err_ of 49.9°, while participants in the Reeder and Corbin studies had mean RMS_err_ of 28° and 29.8°, respectively (Reeder et al. 2015; Corbin et al. 2021). The severity of the hearing loss in the affected ear was poorer for the present sample as compared to previous reports, which may explain the differences in performance. Larger degrees of hearing loss have been associated with poorer localization skills (Reeder et al. 2015; Corbin et al. 2021). There were also substantial individual differences in localization abilities across participant in the SSD group, with performance of 6 participants (01, 08, 11, 14, 15, and 18) approaching the normal range in the CI+NH condition after 24 months of use. Upon examination, it appears that five of these six participants had sudden or progressive loss as opposed to congenital loss. Additional research is needed to understand how hearing history might impact sound source localization in this population.

The current study found a significant influence of age on sound source localization at the 24-month interval for the SSD group in the NH alone condition. Reeder et al. (2015) found a correlation between age and localization for children with UHL but not for children with NH. The authors suggested that children with UHL may require more practice using the subtle localization cues they have access to. While a similar effect could have been at play here, it is also possible that the participants with SSD in the present study learned to use monaural spatial cues as they gained experience with binaural spatial cues, particularly the older participants. In the present study, age at activation had an impact on RMS_err_ with increasing duration of device use, regardless of whether the CI was on or off. In our analyses, the combination of age at activation and test interval was redundant with age at test. Results could therefore indicate that the transfer of binaural skills to the monaural condition may have been easier for the older participants to realize than the younger participants. For example, older children with SSD may be able to use monaural spectral cues to location, as suggested by Corbin et al. (2021).

Constant error early in the follow-up interval was greater for participants implanted at older ages, both with and without the CI. It is possible that the older children had longer preactivation periods of monaural listening experience than the younger participants, leading to early deficits that go away with subsequent binaural exposure; rapid learning might also be supported by prior binaural listening experience. Alternatively, it is possible that older children were more adept at the localization task itself. Research is needed to understand what cues children with SSD use for localization when listening monaurally and binaurally.

### The Impact of CI Use on Auditory Performance for Children With SSD

The results of this prospective clinical trial indicate that cochlear implantation is an effective treatment option for children with SSD, with significant improvements in performance when tested with the CI alone or in bilateral (CI+NH) listening conditions. Previous reports on this cohort have shown that CNC word recognition in the affected ear improved as early as 3 months after activation (Brown et al. 2022). Current findings suggest that the CNC scores have stabilized by 6 months of CI use. After 24 months of CI use, word recognition in the CI ear ranged from 32 to 78% words correct, with a mean of 58%. These findings are very similar to the results of Deep et al. (2021b), who reported a mean CI alone word recognition score of 56% correct for pediatric CI recipients with SSD and significant variability in scores (range, 4–88%). Beck et al. (2017) also found variable improvement in word recognition and attributed the variability to age at implantation; however, the majority of participants in that study had less than 10 months of CI use at the time of testing.

While single-word recognition with the CI alone is often used by clinicians as the primary outcome measure of CI benefit, the overall goal of cochlear implantation in children with SSD is to provide benefits related to binaural listening, specifically spatial hearing. In this study, pediatric participants with SSD demonstrated spatial hearing abilities with CI use. As a whole, BKB-SIN results suggest the use of a CI can improve performance in all tested conditions. The use of a CI improved sentence recognition with colocated and spatially separated target and masker, suggesting benefits of binaural summation, squelch, and head shadow. Whether or not this is truly the effect of binaural hearing is yet to be discovered; however, as some researchers argue that the benefits of the CI in the S_0_N_ci_ condition may be due to redundancy or summation (Dieudonné & Francart 2019). While prospective studies of children with SSD receiving a CI are limited, case studies and retrospective studies also demonstrate the benefits of CI use for masked speech recognition (Plontke et al. 2013; Távora‑Vieira & Rajan 2015; Friedmann et al. 2016; Thomas et al. 2017; Deep et al. 2021a). Similar findings were noted in our earlier reports on this study sample; however, these long-term data suggest that the effects of CI use on hearing in multisource environments for children with SSD are evident as early as 6-month postactivation and are maintained through at least 24 months of bilateral listening experience with a CI (Park et al. 2021a; Brown et al. 2022).

Spatial hearing was also assessed with a sound source localization task. Overall localization error improved over time with the use of a CI, a phenomenon also noted in previous pediatric SSD studies (Hassepass et al. 2013; Plontke et al. 2013; Ehrmann-Mueller et al. 2020; Brown et al. 2022). Interestingly, this improvement was for both the CI+NH and NH alone conditions, indicating that it was not based entirely on acclimatization to the CI. Variable error lessened over time when assessed with CI+NH and remained stable with the NH ear alone. This suggests that children were learning how to use the cues provided by the CI consistently. Constant error was lower with the device on rather than off, indicating that the use of the CI reduced response bias. The use of a CI improved overall localization and response consistency as compared to monaural listening in this cohort.

It should be noted that while a CI improved spatial hearing for both localization and masked speech recognition tasks for all participants, performance was poorer for the SSD group in both conditions than it was for the NH group on all tasks. For masked speech recognition, when the masker was on the side of the CI, performance for the SSD group approached that of the NH group; however, when the masker was presented on the side of the NH-ear performance for the SSD group was poorer than the NH group. While some participants localized as well as peers with NH at the 24-month interval, most did not. At this point, it is unknown whether localization skills will continue to improve beyond the 24-month interval, but it is important to encourage realistic expectations among patients and families. While a CI does provide improvement in spatial hearing, outcomes may not mirror the abilities of children with NH.

An important feature of the present study sample is that most participants in the SSD group were consistent CI users. All participants included in this analysis reported averages of >7 hours of device use per day over the duration of the study. These results are in agreement with other studies that report the majority of pediatric CI users with SSD are consistent users of their devices (Hassepass et al. 2013; Ehrmann-Mueller et al. 2020; Ganek et al. 2020; Rauch et al. 2021). Alternatively, one retrospective study reported limited or nonuse among three of the five participants. Notably, the participants with limited or nonuse of the CI were older than 10 years of age and attributed their lack of use to social stigma (Thomas et al. 2017). Interestingly, the two older children who were excluded from the current report were not full-time CI users. One participant was lost to follow-up and is believed to be a nonuser of their device, and the other reported at the 24-month postactivation interval that they only used their CI in the most challenging listening environments. Both of these participants reported sudden losses ~2 years prior to implantation.

### Effects of Age at Activation

The United States FDA approved cochlear implantation as a treatment option for children with SSD aged 5 years and older in 2019. While future rigorous and well controlled trials are needed to evaluate the impacts of age and length of deafness on CI outcomes in young children with SSD, we do know that earlier age at implantation is beneficial in children with bilateral hearing loss (Geers et al. 2003; Dettman et al. 2007; Niparko et al. 2010; Holman et al. 2013; Leigh et al. 2013; Ching et al. 2018; Karltorp et al. 2020). Recent studies have also found that UHL begins to impact auditory development at very young ages, suggesting that early intervention may be necessary to mediate these effects. For example, Yang et al. (2020) studied infants with UHL at a median age of 4.4 months and found delayed early prelingual auditory skills in infants with SSD as compared to children with NH. Kishon-Rabin et al. (2015) reported delays in the auditory behavior of infants with UHL compared to infants with NH, and Fitzpatrick et al. (2019) noted delays in auditory function in preschool aged children with UHL. It is well known that outcomes for children with bilateral severe-to-profound hearing loss are optimized by early implantation (Leigh et al. 2013, 2016). If UHL begins to impact hearing abilities in infancy, it stands to reason that better outcomes would be experienced with early implantation.

One motivation for evaluating performance in young preschool and school aged children in the present study was the possibility that earlier implantation in cases of SSD would optimize later performance. Imaging and electrophysiological data indicate that that UHL can lead to marked cortical reorganization (Maslin et al. 2013; Heggdal et al. 2016; Han et al. 2021; Calmels et al. 2022), and restoration of hearing can reverse some of these changes and restore binaural function (Polonenko et al. 2017; Karoui et al. 2022). While restoration of binaural hearing can occur in listeners with long-standing hearing loss (Firszt et al. 2013; Távora‑Vieira et al. 2013), hearing loss early in auditory development may be more detrimental than losses acquired later in development (Kral et al. 2015; Tillein et al. 2016; Vanderauwera et al. 2020). Younger age at implantation takes advantage of auditory neuroplasticity, which declines after 7 years of age (Sharma et al. 2002), and may avoid aural preference syndrome, which develops after years of unilateral input (Sharma et al. 2002, 2016; Kral et al. 2013; Tillein et al. 2016; Polonenko et al. 2017, 2018; Lee et al. 2020; Vanderauwera et al. 2020; Anderson et al. 2022).

Recall that the current study sample included children implanted between 3.5 and 6.5 years of age. Comparison of the outcome data at the 24-month interval with data from the NH group provides an opportunity to evaluate the efficacy of implantation as an intervention as a function of age at CI activation, while controlling for known age effects. If earlier implantation leads to better performance, then the difference between SSD and NH groups should grow with increasing age. There was no evidence of a change in the difference between groups for CNC word recognition or for BKB sentence recognition in the three target-to-masker configurations. There was, however, an interaction between group and age for sound source localization; RMS_err_ was elevated in the SSD group relative to the NH group, and this effect was smaller for older children (i.e., those with later age of activation). This effect is opposite of what we might expect if earlier implantation allowed children with SSD to catch up to their peers faster than children with later CI activation. It could be that continued maturation and increased complexity of processing that comes with age is supporting better performance on these complex tasks. One thing to keep in mind is that group differences were evaluated at 24-month postactivation; it is likely that earlier implanted children will catch up to later-implanted children as they continue to develop. It is possible that asymptotic performance will favor earlier implantation, but the data reported here do not indicate any benefit of earlier implantation for children with SSD who were implanted between 3.5 and 6.5 years of age.

### Limitations

While many studies have attempted to find the ideal age for implantation for children with SSD, a recent systematic review has suggested that findings are not yet robust enough to make recommendations (Benchetrit et al. 2021). The present study found significant benefits of CI use for children implanted between the ages of 3.5 and 6.5 years. However, the age span of the participants was rather narrow, limiting our ability to observe age effects. Almost half of the study population had an unknown onset of deafness, precluding a meaningful consideration of the impact of length of deafness on CI outcomes. Unfortunately, this is not unusual for children with UHL and SSD. If hearing loss is not present or detected on newborn hearing screening, it may not be noted until hearing screenings at elementary school enrollment. This is problematic because the incidence grows from 0.1% at birth to 3–6.3% in elementary school (Ross et al. 2010; Shargorodsky et al. 2010).

The present study used a single presentation level of 70 dB SPL for the assessment of sound source localization. While this abbreviated protocol increases feasibility for testing young children, it did not identify the specific cues that pediatric CI users with SSD use for spatial hearing. For example, participants may have used loudness in the NH ear as a localization cue. Ongoing work is evaluating protocols that allow researchers to differentiate reliance on level and timing cues for localization and spatial hearing in this population.

Participants with SSD were tested with their CIs off as an estimate of unaided performance. This may not accurately reflect the performance they would have achieved if they had not received a CI. Removing the CI and testing in a monaural condition could introduce a bias for poorer performance, since children were used to listening with their CI on. Alternatively, it is possible that exposure to binaural listening with the CI could have improved their performance in the monaural condition due to a better understanding of spatial hearing in general. In future studies, children with SSD and no prior binaural experience could be used as a comparison for studies investigating benefits of CI use for cases of SSD.

## CONCLUSIONS

The results of this study indicate that preschool and kindergarten aged children with SSD who receive a CI can experience significant benefits in word recognition in the affected ear, masked sentence recognition and SRM, and sound source localization. While CI use does result in improvement, children with SSD still have poorer performance on these measures than children with NH. Work is needed to evaluate how the significant improvements in spatial hearing and speech recognition observed here influence speech, language, and educational outcomes for this patient population.

## ACKNOWLEDGMENTS

L. R. P., M. T. D., and E. B. are supported by a research grant provided to their university by MED-EL Corporation. K. D. B. serves on the MED-EL and Advanced Bionics surgical advisory boards and is a consultant for Cochlear Corporation. This study was funded by MED-EL Corporation.

## OPEN PRACTICES

This manuscript qualifies for Open Data and Preregistration badges.

The data have been made publically available at https://osf.io/nbmdw,DOI 10.17605/OSF.IO/BPHWF and https://clinicaltrials.gov/ct2/show/NCT02963974. More information about the Open Practices Badges can be found at https://journals.lww.com/earhearing/pages/default.aspx.

## Supplementary Material


