# Supplementary material for: Two-Year Outcomes of Cochlear Implant Use for Children With Unilateral Hearing Loss: Benefits and Comparison to Children With Normal Hearing
Source: Ear Hear. 2023 Mar 7;44(5):955–68. doi: 10.1097/AUD.0000000000001353 (PMC10426784; doi:10.1097/AUD.0000000000001353)
Supplement: Supplementary file 1 [file aud-44-0955-s001.pdf]

Supplemental Digital Content 1. Linear mixed model* results comparing CNC word scores for the SSD NH alone and NH binaural groups when measured in the sound field. The NH group was the reference. The comparison variable is in brackets. Age is centered on 7.0 years, the mean of the SSD group. Testing was completed at the 24-month post-activation interval.

Predictors	*Estimates*	*Conf. Int.*	*p*
(Intercept)	103.34	98.40 – 108.28	**< .001**
Age at test	4.44	0.72 – 8.16	**.026**
Group [SSD]	-15.62	-22.46 – -8.78	**< .001**
Age * Group [SSD]	-1.84	-7.62 – 3.94	.538
Observations	36		
Pseudo R^2	0.396		

Conf. Int., confidence interval; NH, normal hearing; SSD, single-sided-deafness
*Model equation: lme(RAU ~ Age at test * Group, random = ~1|Subject)

Supplemental Digital Content 2. Results of the linear mixed model investigating change over time in CNC word scores measured with the CI alone*. Interval was log-transformed prior to analysis. Age at activation is centered on 5.0 years. Testing was completed at the 3-, 6-, 9-, 12-, 18-, and 24-month post-activation intervals.

Predictors	*Estimates*	*Conf. Int.*	*p*
(Intercept)	-18.10	-28.50 - -7.69	**.001**
Age at activation	8.93	-0.76 - 18.62	.090
Interval	25.21	21.49 - 28.93	**< .001**
Age * Interval	-2.44	-5.91 - 1.03	.170
Observations	108		
Pseudo R^2	0.628		

Conf. Int., confidence interval

*lme(RAU ~ Age at activation * Interval, random = ~1|Subject)

Supplemental Digital Content 3. *Linear mixed model results comparing BKB-SIN SNR-50 scores for the three combinations of group and condition (Group&Cond: SSD CI+NH, SSD NH alone, and NH binaural) for each masker location (MaskerLoc) at the 24-month post-activation interval. The SSD CI+NH and S_0N_0 variables served as references. The comparison variable is in brackets, and age is centered on 7.0 years.

Predictors	*Estimates*	*Conf. Int.*	*p*
(Intercept)	2.79	1.75 – 3.83	**< .001**
Age at test	-0.85	-1.33 – -0.37	**.002**
Group&Cond [SSD NH alone]	1.61	0.52 – 2.69	**.004**
Group&Cond [NH binaural]	-0.14	-1.63 – 1.35	.854
MaskerLoc [$S_0N_{nh/R}$]	-1.25	-2.34 – -0.16	**.026**
MaskerLoc [$S_0Nci_{/L}$]	-3.22	-4.31 – -2.13	**< .001**
Group&Cond [SSD NH alone] * Masker location [$S_0N_{nh/R}$]	2.39	0.85 – 3.93	**.003**
Group&Cond [NH binaural] * MaskerLoc [$S_0N_{nh/R}$]	-4.83	-6.37 – -3.29	**< .001**
Group&Cond [SSD NH alone] * MaskerLoc [$S_0Nci_{/L}$]	-0.14	-1.68 – 1.40	.860
Group&Cond [NH binaural] * MaskerLoc [$S_0Nci_{/L}$]	-2.78	-4.32 – -1.24	**.001**
N_{SubjID}	36		
Observations	162		
Pseudo R^2	0.396		

CI, cochlear implant; Conf. Int., confidence interval; NH, normal hearing; SSD, single-sided-deafness; S_0N_0, speech front masker front; $S_0N_{nh/R}$, speech front masker to the normal hearing or right ear; $S_0Nci_{/L}$, speech front masker to the CI or left ear

* lme(SNR-50 ~ Age at test + Group&Cond * MaskerLoc, random = ~1|Subject)

Supplemental Digital Content 4. Results of comparisons of estimated marginal means derived from the linear mixed model investigating the interaction between combinations of group and condition for each masker location. Bonferroni corrections were used for multiple comparisons.

Masker location	*Comparison*	*Mean Difference (dB)*	*df*	*t-ratio*	*p*
S_0N_0	SSD CI+NH – SSD NH alone	-1.61	119	-2.903	**.013**
	SSD CI+NH – NH binaural	-0.25	33	0.186	1.000
	SSD NH alone – NH binaural	1.36	33	2.306	.083
$S_0N_{nh/R}$	SSD CI+NH – SSD NH alone	-4.00	119	-7.208	**< .001**
	SSD CI+NH – NH binaural	4.58	33	6.548	**< .001**
	SSD NH alone – NH binaural	8.58	33	-11.813	**< .001**
$S_0Nci_{/L}$	SSD CI+NH – SSD NH alone	0.47	119	-2.653	**.027**
	SSD CI+NH – NH binaural	-3.53	33	3.842	**.002**
	SSD NH alone – NH binaural	-2.06	33	5.780	**< .001**

dB, decibel; CI, cochlear implant; NH, normal hearing; SSD, single-sided-deafness; $S_0N_{0,}$ speech front masker front; $S_0N_{nh/R,}$ speech front masker to the normal hearing or right ear; $S_0Nci_{/L,}$ speech front masker to the CI or left ear

Supplemental Digital Content 5. *Linear mixed model results comparing BKB-SIN SNR-50 scores over time for the SSD group in CI+NH and NH alone conditions (Device) in each masker location (MaskerLoc). The NH alone - S_0N_0 condition was the reference. The comparison variable is in brackets, and age at activation is centered on 5.0 years. Testing was completed at the 6-, 12-, and 24-month post-activation intervals.

Predictors	*Estimates*	*Conf Int*	*p*
(Intercept)	5.49	4.57 – 6.41	**< .001**
Age at activation	-0.94	-1.41 – -0.47	**.001**
Interval	-0.04	-0.08 – 0.001	.176
Device[CI+NH]	-1.72	-2.62 – -0.82	**< .001**
MaskerLoc [S_0N_{ci}]	-3.02	-3.92 – -2.12	**< .001**
MaskerLoc [S_0N_{nh}]	0.02	-0.88 – 0.92	.958
Device [CI+NH] * MaskerLoc [S_0N_{ci}]	0.29	-0.98 – 1.547	.657
Device [CI+NH] * MaskerLoc [S_0N_{nh}]	-1.47	-2.74 – -0.20	**.024**
N_{SubjID}	18		
Observations	324		
Pseudo R^2	0.396		

CI, cochlear implant; Conf. Int., confidence interval; NH, normal hearing; SSD, single-sided-deafness; S_0N_{nh}, speech front masker to the normal hearing ear; S_0Nci, speech front masker to the CI ear

* lme(SNR-50 ~ Age at activation + Interval + Device * MaskerLoc, random = ~1|Subject).

Supplemental Digital Content 6. Results of the comparisons of estimated marginal means derived from the mixed model investigating the significant interaction between spatial configuration and CI+NH vs NH alone. Bonferroni corrections were used for multiple comparisons.

	Comparison	*Mean Difference (dB)*	*df*	*t-ratio*	*p*
CI+NH	S_0N_0 - S_0N_{nh}	1.44	300	3.162	**.005**
	S_0N_0 - S_0N_{ci}	2.73	300	5.980	**< .001**
NH alone	S_0N_0 - S_0N_{nh}	-0.02	300	-0.053	1.000
	S_0N_0 - S_0N_{ci}	3.02	300	6.608	**< .001**

CI, cochlear implant; dB, decibel; NH, normal hearing; $S_0N_{0,}$ speech front masker front; $S_0N_{nh/R,}$ speech front masker to the normal hearing or right ear; $S_0Nci_{/L,}$ speech front masker to the CI or left ear

Supplemental Digital Content 7. *Linear mixed model results comparing the log transformed RMS_{err} for the three combinations of group and condition (Group&Cond: SSD CI+NH, SSD NH alone, and NH binaural). The NH binaural group and condition was the reference. The comparison variable is in brackets and age is centered on 7.0 years. Testing was completed at the 24-month post-activation interval.

Predictors	*Estimates*	*Conf Int*	*p*
(Intercept)	2.11	1.95 – 2.27	**<0.001**
Age at test	0.02	-0.10 – 0.14	0.766
Group&Cond [SSD NH alone]	1.82	1.30 – 2.04	**<0.001**
Group&Cond [SSD CI+NH]	0.89	0.67 – 1.11	**<0.001**
Age at test * Group&Cond [SSD NH alone]	-0.23	-0.43 - -0.03	**0.030**
Age at test * Group&Cond [SSD CI+NH]	-0.18	-0.38 – 0.02	0.087
N_{SubjID}	36		
Observations	54		
Pseudo R^2	0.860		

CI, cochlear implant; Conf. Int., confidence interval; NH, normal hearing; SSD, single-sided-deafness

* lme(RMS Error ~ Age at test + Group&Cond , random = ~1|Subject)

Supplemental Digital Content 8. *Results of linear mixed models investigating change in log-transformed RMS_{err} over time for the SSD group measured in the CI+NH and NH alone conditions (Device). The NH alone condition was the reference. The comparison variable is in brackets, and age at activation is centered on 5.0 years. Testing was completed at the 3-, 9-, 18-, and 24-month post-activation intervals.

	**All Test Intervals**		
Predictors	*Estimates*	*Conf. Int.*	*p*
(Intercept)	4.28	4.12 – 4.34	**<0.001**
Age at activation	0.03	-0.1 – 0.17	0.696
Device[CI+NH]	-0.25	-0.42 - -0.07	**0.005**
Interval	-0.01	-0.02 - -0.01	**0.001**
Age at activation * Device[CI+NH]	-0.03	-0.18 – 0.13	0.738
Age at activation * Interval	-0.01	-0.02 - -0.002	**0.006**
Device[CI+NH] * Interval	-0.32	-0.33 - -0.31	**<0.001**
Age at activation * Device[CI+NH] * Interval	< 0.001	-0.009 – 0.01	0.852
N	18_{SubjID}		
Observations	144		
Pseudo R^2	0.781		

CI, cochlear implant; Conf. Int., confidence interval; NH, normal hearing; SSD, single-sided-deafness

* lme(log-transformed RMS error ~ Age at activation * Device* Interval, random = ~1|Subject).

Supplemental Digital Content 9. *Linear mixed model results investigating changes in variable error over time measured in the SSD group CI+NH and NH alone conditions (Device). The NH alone condition was the reference. The comparison variable is in brackets, and age at activation is centered on 5.0 years. Testing was completed at the 3-, 9-, 18-, and 24-month post-activation intervals.

Predictors	*Estimates*	*Conf. Int.*	*p*
(Intercept)	41.09	36.15 – 46.03	**<0.001**
Age at activation	-3.39	-8.00 – 1.22	0.169
Device[CI+NH]	-1.25	-7.05 – 4.55	0.675
Interval	-0.18	-0.43 – 0.75	0.178
Age * Device[CI+NH]	2.89	-2.52 – 8.30	0.297
Age at activation * Interval	0.05	-0.29 – 0.19	0.711
Device[CI+NH] * Interval	-0.88	-1.25 -0.51	**<0.001**
Age at activation * Device [CI+NH] * Interval	-0.18	-0.53 – 0.17	0.297
N_{SubjID}	18		
Observations	144		
Pseudo R^2	0.576		

CI, cochlear implant; Conf. Int., confidence interval; NH, normal hearing; SSD, single-sided-deafness

*lme(Variable error ~ Age at activation * Device* Interval, random = ~1|Subject)

Supplemental Digital Content 10. *Linear mixed model results investigating changes in constant error over time measured in the SSD group CI+NH and NH alone (Group). The NH alone condition was the reference. The comparison variable is in brackets, and age at activation is centered on 5.0 years. Testing was completed at the 3-, 9-, 18-, and 24-month post-activation intervals.

Predictors	*Estimates*	*Conf. Int.*	*p*
(Intercept)	58.40	51.93 – 64.87	**<0.001**
Age at activation	3.43	-2.59 – 9.44	0.281
Group [CI+NH]	-20.45	-28.43 - -12.47	**<0.001**
Interval	-0.65	-1.03 - -0.33	**<0.001**
Age at activation * Group CI+NH]	-4.04	-11.49 – 3.41	0.289
Age at activation * Interval	-0.67	-1.00 - -0.34	**<0.001**
Group [CI+NH] * Interval	-0.37	-0.88 – 0.14	0.157
Age at activation * Group [CI+NH] * Interval	0.53	0.04 – 1.00	**0.029**
N_{SubjID}	18		
Observations	144		
Pseudo R^2	0.674		

CI, cochlear implant; Conf. Int., confidence interval; NH, normal hearing; SSD, single-sided-deafness

*lme(Constant error ~ Age at activation * Group * Interval, random = ~1|Subject)
